# Etiologies of conjugated hyperbilirubinemia in infancy: a systematic review of 1692 subjects

**DOI:** 10.1186/s12887-015-0506-5

**Published:** 2015-11-20

**Authors:** Lena E. Gottesman, Michael T. Del Vecchio, Stephen C. Aronoff

**Affiliations:** Childrens Hospital at Montefiore, New York, NY USA; Department of Pediatrics, Temple University School of Medicine, 3440 N. Broad St., Philadelphia, PA 19104 USA

**Keywords:** Conjugated hyperbilirubinemia, Cholestatic jaundice, Newborn, Differential diagnosis

## Abstract

**Background:**

The etiologies of conjugated hyperbilirubinemia in infancy are diverse.

**Objective:**

Determine the prevalence rates of the specific etiologies of conjugated hyperbilirubinemia in infancy.

**Data sources:**

EMBASE and Pubmed were searched electronically and the bibliographies of selected studies were search manually. The search was conducted independently by two authors.

**Study selection:**

(1) prospective or retrospective case series or cohort study with 10 or more subjects; (2) consecutive infants who presented with conjugated hyperbilirubinemia; (3) subjects underwent appropriate diagnostic work-up for conjugated hyperbilirubinemia; (4) no specific diagnoses were excluded in the studied cohort.

**Data extraction:**

Patient number, age range, country of origin, and categorical and specific etiologies.

**Results:**

From 237 studies identified, 17 studies encompassing 1692 infants were selected. Idiopathic neonatal hepatitis (INH) occurred in 26.0 % of cases; the most common specific etiologies were extrahepatic biliary atresia (EHBA) (25.89 %), infection (11.47 %), TPN- associated cholestasis (6.44 %), metabolic disease (4.37 %), alpha-1 anti-trypsin deficiency (4.14 %), and perinatal hypoxia/ischemia (3.66 %). CMV was the most common infection identified (31.51 %) and galactosemia (36.49 %) was the most common metabolic disease identified.

**Limitations:**

Major limitations are: (1) inconsistencies in the diagnostic evaluations among the different studies and (2) variations among the sample populations.

**Conclusions:**

INH is the most common diagnosis for conjugated hyperbilirubinemia in infancy while EHBA and infection are the most commonly identified etiologies. The present review is intended to be a guide to the differential diagnosis and evaluation of the infant presenting with conjugated hyperbilirubinemia.

## Background

In the newborn period and in early infancy, cholestatic jaundice, or conjugated hyperbilirubinemia, results from hepatobiliary dysfunction. The prevalence of this disorder is estimated at 1 out of every 2500 live births; extrahepatic biliary atresia (EHBA) and idiopathic neonatal hepatitis (INH) account for two thirds of cases of infantile cholestatic jaundice [1].

Most studies of infantile cholestatic jaundice focus on EHBA. Although this disorder is rare (estimated incidence of 1:15,000 live births), timely diagnosis and surgical intervention are required to avoid liver failure and death [1].

Excluding EHBA and INH, a myriad of potential etiologies for infantile cholestatic jaundice have been identified; these can be categorized into obstructive and intrinsic processes. Obstruction can be grossly structural or microscopic in nature (e.g., stones, plugs, or sclerosis of the biliary tract). Intrinsic processes affect hepatic cellular function (e.g. infections, metabolic disorders, endocrinopathies, chromosomal abnormalities, vascular abnormalities, toxins, neoplasms, and prematurity) [2]. Given the absence of large, comprehensive patient series that define the spectrum and relative incidence of the etiologies of cholestasis in the newborn period and early infancy, this systematic review was undertaken.

## Methods

### Ethics

This study involved the use of published, summary data from the world’s literature. There was no contact with primary, patient- identified, source data. Temple University's Human Research Protection Program has declared that evaluations of publically available, secondary databases of de-identified patient data are not subject to its review.

### Protocol

This study followed the PRISMA guidelines [3].

### Eligibility criteria

The study protocol was developed by the authors *a priori*. The inclusion criteria for this review were:

1. prospective or retrospective case series or cohort study with 10 or more subjects;
2. consecutive infants who presented with conjugated hyperbilirubinemia defined as elevations of total and direct serum bilirubin concentrations beyond the immediate newborn period;
3. appropriate diagnostic work-up for conjugated hyperbilirubinemia and;
4. no specific diagnoses were excluded in the studied cohort.

### Search

The main search terms were “conjugated hyperbilirubinemia” or “cholestatic jaundice” and “neonates” or “infants.” The following filters were used: human. No language filter was used; however, when abstract review was required, citations without English abstracts were eliminated. The searches were performed independently by two of the authors and the results were compared.

### Study selection

Initial evaluation of each article was performed by author LEG and then reviewed by author MTD. Differences in judgment were resolved first by consensus; ties were adjudicated by the third author (SCA).

### Data collection

For each selected study, the following information was recorded: number of patients, age range of patients, and country of origin. The categorical and specific etiologies of conjugated hyperbilirubinemia and the number of patients classified into each group for all selected studies were recorded. The categories of disease included EHBA, INH, total parenteral nutrition (TPN)—associated cholestasis, Alagille’s syndrome, alpha 1-antitrypsin deficiency, cystic fibrosis (CF), infection, hypopituitarism/hypothyroidism, progressive familial intrahepatic cholestasis (PFIC), perinatal hypoxia/ischemia, interlobular bile duct paucity, inspissated bile syndrome, choledochal cysts, hemolysis, metabolic diseases, and others. Within the categories of infection and metabolic diseases, specific etiologies were catalogued based on the data provided in each study. Data collection was reviewed by the second author (MTD).

### Synthesis of results

Results from all of the studies were pooled to provide rate estimates of categorical and specific etiologies of cholestasis. Rate estimates for etiologic categories were calculated from the number of subjects used in the entire review. Rate estimates for specific infectious and metabolic etiologies used the total number of subjects reported with specific infectious and metabolic disorders as the denominator.

### Sources of bias across studies

Variability in diagnostic evaluations and the potential for overestimation of INH represent possible sources for bias. The identification of multiple etiologies in individual patients as well as inconsistencies and vagaries in nomenclature were potential sources of error when studies were combined. In some cases, delayed clinical presentation may have affected diagnostic accuracy.

## Results and discussion

### Study selection

**Fig. 1:**
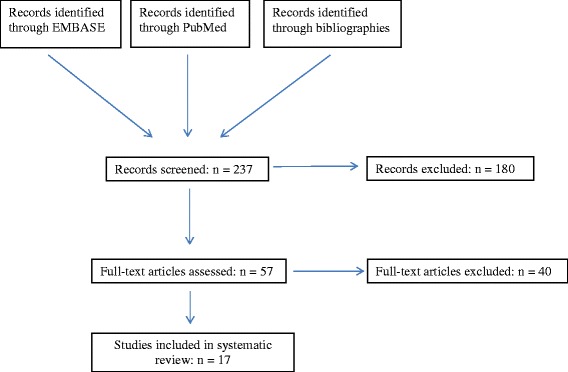
Summary of Literature Search

The results of the literature search are shown in the Fig. 1. Searches of Medline and EMBASE databases yielded 193 references. An additional 44 citations were found by extensively searching the bibliographies of selected articles. From the 237 studies identified, 180 studies were excluded after a cursory review of the title, abstract, and, when necessary, the results section. The full text of the remaining 57 articles was reviewed in detail. Forty of the remaining studies were excluded: 11 reports failed to report specific diagnoses; 6 reports had inclusion criteria that were too narrow; 6 reports included patients without conjugated hyperbilirubinemia; 6 reports were not case series or cohort studies; 5 reports had subjects with previously identified disease processes; 2 failed to include a detailed clinical evaluation; 1 each had vague inclusion criteria, non-consecutive patients, included the same patient population from another selected study, or had a sample size of less than 10. The remaining 17 studies comprise this review [1, 4–20].

### Study characteristics and outcomes

The 17 studies that met the inclusion criteria are presented in Table 1 [1, 4–20]. These reports ranged in size from 20 to 249 participants and represented a worldwide sample (United States, Turkey, United Kingdom, Bangladesh, China, Sweden, South Africa, Iran, Nigeria, Australia, India, and Thailand). Children were drawn from single centers in 15 studies and entire regions in 2 studies. The number of patients included in this review is 1692.

**Table 1:** Summary of included studies

Study citation	# of patients	Age range of patients at presentation	Country of origin
Hitch et. al [19]	28	1.5–17 weeks	United States
Tolia et. al [12]	20	2–28 weeks	United States
Tiker et. al [13]	42	0–4 weeks	Turkey
Humphrey et. al [18]	90	1–21 weeks	United Kingdom
Bazlul Karim et. al [7]	62	4–92 weeks	Bangladesh
Jiang et. al [11]	50	4–24 weeks	China
Fischler et. al [10]	85	1–39 weeks	Sweden
Spivak et. al [14]	33	Not recorded	United States
Motala et. al [15]	145	Not recorded	South Africa
Ipek et. al [17]	92	0–10 weeks	Turkey
Rafeey et. al [4]	122	2–17 weeks	Iran
Johnson et. al [16]	101	0–24 weeks	Nigeria
Stormon et. al [9]	205	0–24 weeks	Australia
Mowat et. al [8]	137	0–12 weeks	England
Yachha et. al [6]	60	8–24 weeks	India
Aanpreung et. al [5]	249	0–12 weeks	Thailand
Danks et. al [20]	171	0–6 weeks	Australia

The etiologies of infantile conjugated hyperbilirubinemia, by study, are shown in Table 2. Humphrey et. al reported 15 subjects with “prematurity, sepsis and parenteral nutrition” [18]. These subjects were grouped into the “other” category because a single etiology could not be chosen. Tolia et. al. excluded subjects whose cholestatic jaundice resolved after 6 months and thus did not undergo a full diagnostic work-up [12]. Johnson et. al excluded nine subjects with biliary tract obstruction but “were either too ill or parents declined ex-lap to define nature” and, eight undiagnosed subjects who did not return for re-evalaution [16]. All 17 of these patients were included in the present review and categorized as “other”. Spivak excluded five subjects who “did not have scans because they either were too ill to transport or died before the study” [14]. These subjects were included and classified as “other.” Motala et. al excluded TPN- associated cholestasis and Danks et. al excluded subjects with choledochal cysts [15, 20]. Attempts to contact these authors were unsuccessful. Despite exclusions of specific diagnoses, these studies were retained.

**Table 2:** Etiology of conjugated hyperbilirubinemia in infancy by study

	Extrahepatic BA	Ideopathic Neonatal Hepatitis	TPN associated cholestasis	Alagille Syndrome	Interlobular bile duct paucity	Alpha 1 Antitrypsin deficiency	Cystic Fibrosis	Infection	Hypopituitarism hypothyroid	Progressive familial intrahepatic cholestasis	Perinatal hypoxia-ischemia	Inspissated bile syndrome	Choledochal cyst	Hemolysis	Metabolic disease	Other ^a^
	N/%	N/%	N/%	N/%	N/%	N/%	N/%	N/%	N/%	N/%	N/%	N/%	N/%	N/%	N/%	N/%
Hitch et. al [18]	9	7	6	-	1	1	-	-	-	-	-	-	1			3
	32.14%	25.00%	21.43%		3.57%	3.57%							3.57%			10.71%
Tolia et. al [11]	6	2	6	-	2	1	-	2	-	-	-	-	-	-	-	1
	30.00%	10.00%	30.00%		10.00%	5.00%		10.00%								5.00%
Tiker et. al [12]	1	2	3	-	-	-	-	16	1	-	7	-	-	5	1	6
	2.38%	4.76%	7.14%					38.10%	2.38%		16.67%			11.90%	2.38%	14.29%
Humphrey et. al [17]	30	22	-	4	2	4	3	2	2	2	-	1	1	-	-	17
	33.33%	24.44%		4.44%	2.22%	4.44%	3.33%	2.22%	2.22%	2.22%		1.11%	1.11%			18.89%
Bazlul Karim et. al [5]	16	15	-	-	1	1	-	22	3	-	-	-	4	-	-	-
	25.81%	24.19%			1.61%	1.61%		35.48%	4.84%				6.45%			
Jiang et. al [10]	23	22	-	-	-	-	-	-	-	1	-	-	2	-	2	-
	46.00%	44.00%								2.00%			4.00%		4.00%	
Fischler et. al [9]	30	15	-	3	-	11	1	7	1	11	-	-	-	-	-	6
	35.29%	17.65%		3.53%		12.94%	1.18%	8.24%	1.18%	12.94%						7.06%
Spivak et. al [13]	7	6	5	-	6	-	-	1	-	-	-	3	-	-	-	5
	21.21%	18.18%	15.15%		18.18%			3.03%				9.09%				15.15%
Motala et. al [14]	41	52	Excludeds^♦^	-	-	6	1	35	-	-	-	-	5	-	5	-
	28.28%	35.86%				4.14%	0.69%	24.14%					3.45%		3.45%	
Ipek et. al [16]	4	7	8	-	2	-	-	9	4	-	28	6	-	15	6	3
	4.35%	7.61%	8.70%		2.17%			9.78%	4.35%		30.43%	6.52%		16.30%	6.52%	3.26%
Rafeey et. al [2]	30	44	-	1	13	-	1	4	-	1	-	-	2	-	25	1
	24.59%	36.07%		0.82%	10.66%		0.82%	3.28%		0.82%			1.64%		20.49%	0.82%
Johnson et. al [15]	31	33	-	-	-	1	-	12	-	-	-	3	2	-	-	19
	30.69%	32.67%				0.99%		11.88%				2.97%	1.98%			18.81%
Stormon et. al [7]	34	18	35	6	1	13	7	23	10	2	21	9	3	-	17	6
	16.59%	8.78%	17.07%	2.93%	0.49%	6.34%	3.41%	11.22%	4.88%	0.98%	10.24%	4.39%	1.46%		8.29%	2.93%
Mowat et. al [6]	32	61	-	-	1	24	2	9	-	-	-	-	2	-	2	4
	23.36%	44.53%			0.73%	17.52%	1.46%	6.57%					1.46%		1.46%	2.92%
Yachha et. al [4]	33	7	-	-	2	-	-	5	-	-	-	-	-	-	2	11
	55.00%	11.67%			3.33%			8.33%							3.33%	18.33%
Aanpreung et. al [3]	56	58	46	2	-	-	-	25	12	-	6	1	14	4	8	17
	22.49%	23.29%	18.47%	0.80%				10.04%	4.82%		2.41%	0.40%	5.62%	1.61%	3.21%	6.83%
Danks et. al [19]	55	69	-	-	11	8	-	22	-	-	-	-	Excluded^✧^	-	6	-
	32.16%	40.35%			6.43%	4.68%		12.87%							3.51%	

^a^ See text for complete list and count of “other” diagnoses

♦TPN associated cholestasis was excluded from Motala et. al. See text for further detail.

✧Choledochal cysts were excluded from Danks et. al. See text for further detail

### Synthesis of results

The etiologies of conjugated hyperbilirubinemia in infancy were defined categorically, by process, and by specific disease entity, where adequate data existed. The categorical etiologies are shown in Table 3. Of the 1692 subjects who comprise this review, INH was reported in 440 (26.0 %); EHBA occurred in 438 subjects (25.9 %) and infection was identified in 194 subjects (11.5 %). Less common categorical causes of infantile cholestatic jaundice included: TPN -associated cholestasis (109 subjects, 6.4 %), metabolic disease (74 subjects, 4.4 %), alpha-1 antitrypsin deficiency (70 subjects, 4.1 %), perinatal hypoxia/ischemia (62 subjects, 3.7 %), interlobular bile duct paucity (42 subjects, 2.5 %), choledochal cyst (36 subjects, 2.1 %), hypopituitarism/hypothyroidism (33 subjects, 2.0 %), hemolysis (24 subjects, 1.4 %), inspissated bile syndrome (23 subjects, 1.4 %), PFIC (17 subjects, 1.0 %), Alagille syndrome (16 subjects, 1.0 %), and cystic fibrosis (15 subjects, 0.9 %). Diagnoses categorized as “other” occurred in 99 subjects (5.9 %) and are listed in Table 4.

**Table 3:** Summary of etiologies of conjugated hyperbilirubinemia in infancy by disease category

	Total number	% of total
Idiopathic Neonatal Hepatitis (INH)	440	26.00 %
Extrahepatic Biliary Atresia (EHBA)	438	25.89 %
Infection	194	11.47 %
TPN associated cholestasis	109	6.44 %
Metabolic disease	74	4.37 %
Alpha-1 Antitrypsin deficiency	70	4.14 %
Perinatal hypoxia/ischemia	62	3.66 %
Interlobular bile duct paucity	42	2.48 %
Choledochal cyst	36	2.13 %
Hypopituitarism/hypothyroidism	33	1.95 %
Hemolysis	24	1.42 %
Inspissated bile syndrome	23	1.36 %
Progressive Familial Intrahepatic Cholestasis (PFIC)	17	1.00 %
Alagille syndrome	16	0.95 %
Cystic Fibrosis	15	0.89 %
Other ^a^	99	5.85 %
Total	1692	100.00 %

^a^ See text for complete list and count of “other” diagnoses

**Table 4:** Other etiologies of conjugated hyperbilirubinemia

	Total number	% of total
Icterus of unknown cause	19	19.19 %
Trisomy 21	16	16.16 %
Cholestasis of prematurity	15	15.15 %
Sujcts too ill to transport to scanner, died before the study, or parents declined diagnostic procedures	15	15.15 %
Undiagnosed subjects who did not return for follow-up	8	8.08 %
Neonatal systemic lupus erythematosus	5	5.05 %
Mitochondrial dysfunction	3	3.03 %
Neonatal sclerosing cholangitis	2	2.02 %
Common bile duct (CBD) stones	2	2.02 %
Congenital hepatic fibrosis	1	1.01 %
Portal venous thrombosis	1	1.01 %
Aagenae’s syndrome	1	1.01 %
Carbohydrate deficient glycoprotein	1	1.01 %
Familial hemophagocytic lymphohistiocytosis	1	1.01 %
Annular pancreas	1	1.01 %
Arthrogryposis syndrome	1	1.01 %
Histiocytosis X	1	1.01 %
Stenosis of the choledochojejunal junction	1	1.01 %
Hydrocephalus	1	1.01 %
Cleidocranial dysostosis	1	1.01 %
Cardiomyopathy/hydrops fetalis	1	1.01 %
Renal tubular acidosis	1	1.01 %
Spontaneous perforation of the CBD	1	1.01 %
Total	99	100.00 %

The specific infectious etiologies associated with infantile conjugated hyperbilirubinemia are shown in Table 5. Among the 194 subjects with an infectious etiology, CMV was identified in 65 subjects (33.5 %). Sepsis (24.7 %), congenital syphilis (10.8 %), and E. coli UTI (9.8 %) were the next most common entities identified. Of the patients with sepsis, bacterial and viral etiologies were identified in 11: *Pseudomonas aeruginosa*, *Staphylococcus* species, *Klebsiella* species, *E. coli*, cocksackie B, and parainfluenza type 3 [16, 17, 20].

**Table 5:** Infectious causes of conjugated hyperbilirubinemia in infancy

	Total number	% of total
CMV	65	33.51 %
Sepsis ^a^	48	24.74 %
Congenital Syphilis	21	10.82 %
E. coli UTI	19	9.79 %
Rubella	12	6.19 %
Toxoplasmosis	7	3.61 %
Hepatitis B	3	1.55 %
Herpes Simplex	2	1.03 %
Other ^b^	17	8.76 %
Total	194	100.00 %

^a^ See text for explanation of sepsis

^b^ EBV, cholangiolitis, Klebsiella UTI, Enterovirus (3), Tuberculosis, hemophagocytic syndrome, HIV (3), Candidemia (3), Pneumonia (2), unknown (2)

Metabolic disorders associated with infantile conjugated hyperbilirubinemia are shown in Table 6. Among the 74 subjects reported to have a metabolic disease, galactosemia was identified in 27 subjects (36.5 %). Thirteen subjects (17.6 %) had undefined metabolic disease. Glycogen storage disease, tyrosinemia, and iron storage disease accounted for 9.5, 8.1, and 8.1 % respectively.

**Table 6:** Metabolic disease as causes of conjugated hyperbilirubinemia in infancy

	Total number	% of total
Galactosemia	27	36.49 %
Glycogen Storage Disease	7	9.46 %
Tyrosinemia	6	8.11 %
Iron Storage Disease	6	8.11 %
Niemann-Pick	4	5.41 %
Zellweger	3	4.05 %
Fat storage disease	2	2.70 %
Hereditary fructose intolerance	2	2.70 %
HMG CoA lyase deficiency	1	1.35 %
Citrullinemia	1	1.35 %
Methyl-malonic acidemia	1	1.35 %
Gaucher disease	1	1.35 %
Unknown*	13	17.57 %
Total	74	100.00 %

*See text for complete list and count of “unknown” diagnoses

### Risk of bias across studies

Study sizes ranged from 20 to 249 subjects; the largest study accounted for 14.7 % of the total sample reducing the risk of selection bias in the pooled results. The subjects represented 12 countries and five continents; 2 studies drew patients from entire regions and the remaining studies each represented one clinical site. Eight of the centers were referral sites. Nine of the studies were prospective and eight were retrospective. Five studies focused on specific diagnostic techniques to differentiate biliary atresia from neonatal hepatitis.

Tiker et. al and Ipek et. al studied subjects admitted to neonatal intensive care units [13, 17]. In Tiker et. al, etiologic prevalence rates differed from those of the other studies presumably due to the study’s narrow inclusion of newborns less than 1 month of age [13].

Vagaries associated with terminology, e.g., “neonatal hepatitis,” “idiopathic neonatal hepatitis,” and “cholestatic jaundice,” were encountered in multiple studies [6, 12, 13]. For the purpose of this review, patients were categorized into idiopathic neonatal hepatitis if no underlying etiology was found. Yachha et. al classified seven subjects as neonatal hepatitis and 11 as neonatal cholestatic syndrome of indeterminate etiology; [6] neonatal hepatitis subjects were re-classified into INH and the rest were re-classified as “other” in this review.

Concurrent diagnoses were also a source of bias across studies. Ipek et. al categorized each subject under a single etiology but reported that “the majority of infants (80.4 %) had concomitant clinical disorders that might have contributed to the development of conjugated hyperbilirubinemia.” [17] Aanpreung et. al cited 46 subjects with TPN—associated cholestasis but reported that 41 of these infants were premature and “TPN was not a single cause since there could be other causes such as hypoxia, sepsis, and drug-induced” [5]. These 46 subjects were categorized under TPN- associated cholestasis since the author chose to identify them as such. Similarly, Humphrey et. al reported 15 subjects with “prematurity with sepsis and parenteral nutrition” [18]. Since this incorporated multiple diagnoses without a single diagnosis favored, these subjects were classified as “other.” Tolia et. al categorized one subject as both neonatal hepatitis and TPN associated cholestasis [12]. This subject was re-categorized under TPN associated cholestasis.

Although Downs syndrome is not a proven cause of conjugated hyperbilirubinemia, it was cited as the etiology in 4 studies [5, 8, 10, 13]. In these cases, if multiple diagnoses were identified, the most probable cause was used to categorize the subject; if Downs syndrome was the sole diagnosis, the subject was categorized as “other.” Tiker et. al identified Downs syndrome as the etiology in three subjects, 2 of whom had concurrent diagnoses of hypothyroidism and idiopathic neonatal hepatitis [13]. These 2 subjects were assigned to categories based on the concurrent diagnosis. Fischler et. al cited Downs syndrome as a sole diagnosis in one subject [10]. Mowat et. al identified “chromosomal trisomy” as “possibly but not definitely causative” of neonatal hepatitis in four subjects who did not have evidence of other etiologies [8]. These four subjects were classified as “other.” Aanpreung et. al cited Downs syndrome as the sole etiology in 11 subjects [5].

### Limitations

Infantile conjugated hyperbilirubinemia presents with persistent jaundice as part of a clinical constellation that may include other symptoms based on the underlying etiology. The differential diagnosis is broad and requires timely evaluation [2]. The data presented in this review suggest that INH, EHBA, and infection (with CMV being the most common infection) account for 63.36 % of all cases of infants presenting with elevated serum concentrations of conjugated bilirubin.

The diagnostic evaluation should be guided by symptomatology and may include various imaging studies and serologic, hematologic, and urine investigations for various infections and endocrinopathies, as well as genetic testing for inborn errors of metabolism. While the definitive diagnosis of EHBA requires a percutaneous liver biopsy, ongoing research is investigating less invasive methods of differentiating EHBA from other etiologies of infantile conjugated hyperbilirubinemia [1].

Inconsistency of the diagnostic approach is a major limitation of this review. While there are general guidelines directing the evaluation of an infant with conjugated hyperbilirubinemia, reports published prior to establishment of these guidelines are included in the present review [1]. Moreover, diagnostic practices vary by country and multiple studies focused on the ability of a specific radiologic test to differentiate EHBA from other causes of conjugated hyperbilirubinemia. Together, these conditions may introduce inherent disparities in evaluation and may contribute to bias among these studies [11, 12, 14, 18, 19].

Variability in sample populations is also a potential source of bias. While 12 countries and 5 continents are represented, there is no data from Eastern Europe or South America. Disorders that may be uniquely prevalent in these areas may be underrepresented. Finally, summary data that includes subjects from all over the world may be less relevant to any specific country given the uneven distribution of etiologies between developed and developing countries as well as diseases endemic to the East and West.

Finally, the use of the category of INH to include all idiopathic cases of infantile conjugated hyperbilirubinemia is a potential source of bias in this review [6, 10–14, 16, 18, 19]. While Ipek et. al defined INH as conjugated hyperbilirubinemia that persists beyond 3 months without another identifiable cause [17], multiple studies did not specifically use the term INH or provide a definition.

## Conclusions

The etiologies of infantile conjugated hyperbilirubinemia are numerous. Because of the consequences of untimely correction of EHBA as well as the potential consequences of untreated galactosemia, hypothyroidism and other etiologies associated with this problem, clinical evaluation needs to be prompt, focused, and complete. While specific symptoms may narrow the diagnostic possibilities, a complete history and physical examination, diagnostic imaging and laboratory investigations directed at the more common etiologies is required to make a prompt, definitive diagnosis. This systematic review provides evidence to direct the investigation of an infant with conjugated hyperbilirubinemia.
